# Chlorinated Paraffins in Chicken Eggs from Five Regions in China and Dietary Exposure Health Risk Assessment

**DOI:** 10.3390/toxics14010060

**Published:** 2026-01-08

**Authors:** Nan Wu, Lei Zhang, Tingting Zhou, Jiyuan Weng, Changliang Li, Wenjie Song, Yingying Zhou, Qi Li, Yu Lu, Pingping Zhou, Lirong Gao

**Affiliations:** 1 NHC Key Laboratory of Food Safety Risk Assessment, China National Center for Food Safety Risk Assessment, Beijing 100022, China; 2 State Key Laboratory of Environmental Chemistry and Ecotoxicology, Research Center for Eco-Environmental Sciences, Chinese Academy of Sciences, Beijing 100085, China

**Keywords:** chlorinated paraffins, chicken eggs, dietary exposure, risk assessment

## Abstract

Chlorinated paraffins (CPs) are a class of persistent organic pollutants that pose potential human health risks through dietary exposure. In this study, we analyzed CPs in 55 chicken egg samples collected from five regions across China. Short-chain chlorinated paraffins (SCCPs) and medium-chain chlorinated paraffins (MCCPs) were detected using a two-dimensional gas chromatograph coupled with an electron-capture negative-ionization mass spectrometer. Dietary exposure risks were assessed using the margin of exposure (MOE) approach based on the food consumption data of Chinese residents from 2018 to 2020. The average concentrations of SCCPs and MCCPs in all samples were 28.4 ng/g wet weight (ww) and 176.5 ng/g ww, respectively. The congener profiles of SCCPs and MCCPs were similar across different regions, with C_10–11_ Cl_6–7_ as the dominant homologs. For MCCPs, the average contributions of C_14_-CP, C_15_-CP, C_16_-CP, and C_17_-CP were 25%, 21%, 27%, and 27%, respectively. The estimated daily intake (EDI) for the entire population was 18.3 ng/kg body weight (bw)/d for SCCPs and 118.3 ng/kg bw/d for MCCPs. In the consumer-only group, the average exposure levels of SCCPs and MCCPs were 27.8 ng/kg bw/d and 174.1 ng/kg bw/d, respectively. This preliminary risk assessment indicates that there is no health risk to the Chinese population from exposure to CP through consumption of chicken eggs.

## 1. Introduction

Chlorinated paraffins (CPs) are mixtures of polychlorinated alkanes and are categorized by carbon chain length into short-chain CPs (C_10–13_, SCCPs), medium-chain CPs (C_14–17_, MCCPs), and long-chain CPs (C > 17, LCCPs) [1]. The general chemical structures of CPs are shown in Figure S1. CPs are widely used as plasticizers, lubricants, sealants, metalworking fluids, and flame retardants in industrial fields [2,3]. China is a major global producer and consumer of CPs [4]. CPs can contaminate the environment in various ways during production and transportation [5,6,7,8]. CPs can bioaccumulate and bioamplify through the food chain, especially in aquatic ecosystems. The bioaccumulation coefficient is affected by factors such as carbon chain length, degree of chlorination, and species, leading to elevated concentrations in organisms with higher trophic levels [9]. Furthermore, CPs have been detected not only in fish, birds, and other mammals, but also in human blood and breast milk [7,10,11,12]. Clearly, CPs inevitably transfer to food, where they accumulate after being inadvertently released into the environment [13]. Dietary intake is the main route of human exposure to CPs [14].

There is increasing concern worldwide about SCCPs due to their potential hazards compared to the other CP mixtures [15]. The acute toxicity of SCCPs in animals is low; however, they possess potential carcinogenicity [16]. The International Agency for Research on Cancer (IARC) classified SCCPs (C_12_, 60% chlorinated) as Group 2B carcinogens in 1990 [17]. Studies on animals suggest that SCCPs primarily affect the liver, kidneys, thyroid, and parathyroid glands [18,19,20]. Furthermore, exposure to even low doses of SCCPs can induce metabolic disorders in rats [21]. Given the persistent organic pollutants (POPs) nature of SCCPs, there is increasing international consensus advocating for their restrictive use. In 2017, SCCPs were recognized as POPs and listed in Annex A (Elimination) of the Stockholm Convention [22]. Furthermore, MCCPs and SCCPs exhibit similar structures and demonstrate comparable cytotoxicity and metabolic perturbation effects [23]. In 2025, MCCPs were listed in Annex A of the Stockholm Convention [24].

Although some achievements have been made in understanding the harmfulness of CPs, studies on exposure assessment or toxicity determination are limited by the quantification of CPs due to their low response in various detection systems and their highly complex nature [25,26]. The log Kow of CPs ranges from 4 to 12, suggesting that they are highly lipophilic and bioaccumulative chemicals [27]. Several studies have shown that CPs can be detected in a variety of foods, including meat, grain, seafood and eggs [28,29,30]. In the sixth national total dietary survey in China, researchers found that the concentration of CPs in most animal-derived foods was higher than that in plant-derived foods. The highest concentrations of SCCPs and MCCPs were found in meat, followed by eggs [31]. Studies have shown that chickens can accumulate CPs from feed, transferring them from chicken tissues to eggs. The accumulation rate in chickens increases with the carbon chain length and chlorine substitution number [32]. As an important component of people’s daily diet, eggs can reflect the bioaccumulation of CPs and the level of human exposure to CPs through egg intake [33]. Chicken eggs constitute a significant portion of the food consumption among the Chinese population, while the extent of contamination is unclear.

As an important exposure pathway in daily life, diet is of great significance in assessing the risk of exposure to CPs. To assess the health risks associated with dietary intake of CPs in the population, it is crucial to further understand the contamination characteristics and distribution of CPs in foods. We collected raw chicken egg samples from the market across five different regions of China, and determined the levels of SCCPs and MCCPs. Our objectives were to understand the status of SCCPs and MCCPs contamination in eggs from different regions, the characteristics of their homologous groups, and to assess the health risks of exposure to SCCPs and MCCPs through egg consumption in the Chinese population.

## 2. Materials and Methods

### 2.1. Sample Collection

**Figure 1:**
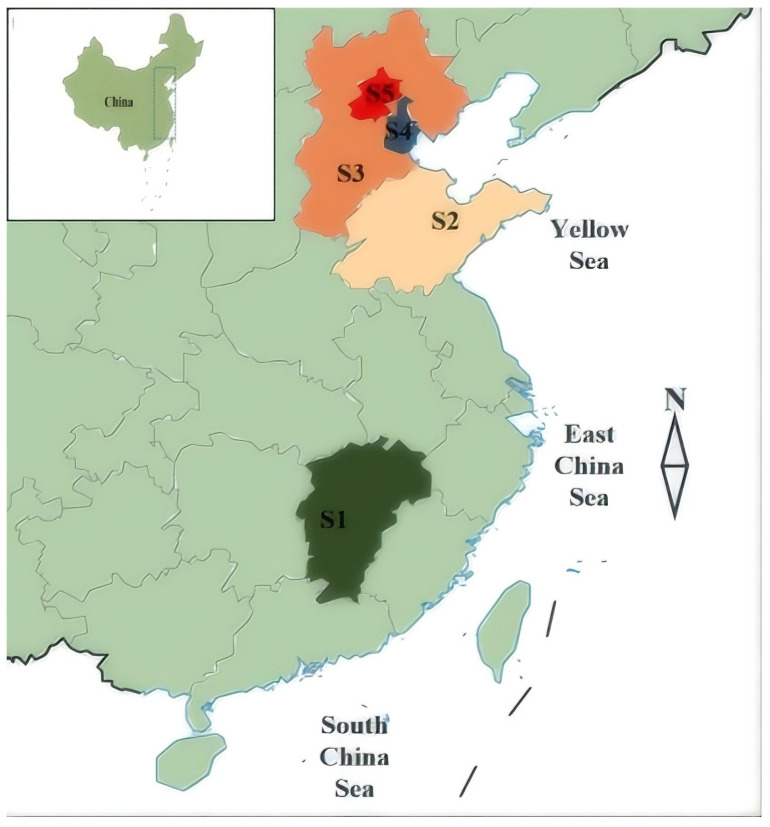
Sampling locations in five regions of China (S1: Jiangxi; S2: Shandong; S3: Hebei; S4: Tianjin; S5: Beijing).

To investigate the contamination status of CPs in chicken eggs from different regions of southern and northern China, this study selected five areas. A total of 55 chicken egg samples were collected from supermarkets in three provinces and two municipalities in 2023. The chicken egg samples were purchased from supermarkets in Jiangxi (11 samples), Shandong (10 samples), Hebei (15 samples), Tianjin (9 samples), and Beijing (10 samples). Hebei, Shandong, Tianjin, and Beijing are located in northern China, while Jiangxi is a province in southern China. Considering that southern China’s economy is more developed and industrialized. In this study, the sample selection took into account regional differences across China (see Figure 1).

### 2.2. Reagents and Materials

The organic solvents used for extraction and purification (n-hexane, acetone, cyclohexane, and dichloromethane) were of pesticide grade and were purchased from JT Baker (Phillipsburg, NJ, USA). Analytical-grade reagents, including methanol, acetone, and dichloromethane, used for rinsing glassware prior to experiments, were supplied by Sinopharm Chemical Reagent Co., Ltd. (Shanghai, China). Standards (100 ng/μL in cyclohexane) of SCCPs (51.5%, 55.5%, and 63% chloride content) and MCCPs (42%, 52%, and 57% chloride content), as well as the internal standard ε-hexachlorocyclohexane (10 ng/μL), were obtained from Dr. Ehrenstorfer (Augsburg, Germany). The internal standard, ^13^C_10_-trans-chlordane (100 ng/μL), was purchased from Cambridge Isotope Laboratories (Tewksbury, MA, USA). All filler materials were conditioned by baking in a muffle furnace. Silica gel (63–100 μm; Merck KGaA, Darmstadt, Germany) and Florisil (60–100 mesh; Merck KGaA) were baked at 550 °C for 6.5 h and 12 h, respectively. Anhydrous sodium sulfate (Kemiou Chemical Reagent Company, Tianjin, China) was heated at 650 °C for 6.5 h. Acidified silica gel was prepared by mixing baked activated silica gel with concentrated sulfuric acid at a ratio of 44% sulfuric acid by mass. Further details on the chemicals and standards used in this study are provided in Table S1.

### 2.3. Sample Preparation and Analysis

Approximately 5 g of freeze-dried egg samples were spiked with 2.5 ng of ^13^C_10_-trans-chlordane. The samples were extracted with an ASE350 accelerated solvent extractor (Dionex, Sunnyvale, CA, USA) with 150 mL of a dichloromethane/n-hexane (1:1, *v*/*v*). The extract was rotary evaporated to near-dryness, dissolved in 60 mL of n-hexane, and treated with 30 g of 44% acidified silica gel to remove lipids. The supernatant was then collected and transferred. The acidified silica gel was rinsed with 30 mL of n-hexane, followed by two additional rinses with 15 mL of a n-hexane/dichloromethane mixture (1:1, *v*/*v*). All supernatants were combined and concentrated by rotary evaporation to a final volume of 1–2 mL. Finally, 2.5 ng of ε-hexachlorocyclohexane was added to the extract before instrumental analysis. A two-dimensional gas chromatograph coupled with an electron-capture negative-ionization mass spectrometer (Agilent Technologies, Santa Clara, CA, USA) was used to analyze the concentrations of SCCPs and MCCPs in the extracts after pre-treatment. The instrument settings are available in the Supplementary Material (Text S2), and the quantitation and qualification ions for SCCP and MCCP determination are listed in Table S2.

### 2.4. Quality Assurance and Quality Control

Quality assurance and quality control (QA/QC) measures were rigorously implemented throughout the analytical workflow to ensure the reliability of the results. All glassware was rinsed three times sequentially with anhydrous methanol, acetone, and dichloromethane prior to use to minimize potential background interference. To assess contamination by SCCPs and MCCPs, a procedural blank was included in each batch of twenty samples. Procedural blanks were prepared following the same protocol as the egg samples, except that clean diatomaceous earth was used in place of the actual food matrix during extraction. Most SCCP and MCCP congener groups in the procedural blanks were below the detection limit. The only exceptions were the C_10_Cl_5–7_ and C_11_Cl_5–6_ congener groups, which were detected at concentrations less than 10% of the minimum quantifiable level in real samples. The method detection limit (MDL) for SCCPs was defined as the mean concentration of SCCPs in the blanks plus three times the relative standard deviation (RSD), yielding a value of 6 ng/g. For MCCPs, the MDL was determined by spiking blank samples with MCCPs at 20 ng/g and performing seven parallel determinations; based on the RSD of these replicate analyses, the MDL for MCCPs was calculated to be 4 ng/g. Parallel samples were analyzed in each batch of twenty samples, and the RSD values were all below 20%. Additionally, recoveries of the surrogate standard (^13^C_10_-trans-chlordane) across all samples ranged from 75% to 112%. Representative ECNI–MS spectra and total ion chromatograms are provided in the Supplementary Material (Figures S2–S7).

### 2.5. Food Consumption Data

The data on chicken egg consumption were obtained from a dietary survey conducted in 19 provinces and four municipalities in China between 2018 and 2020. The survey employed 24 h retrospective recall, food frequency method and food weighing method to assess the dietary intake of residents aged over 3 years. The egg consumption data included raw, cooked, and poached eggs. A total of 55,700 individual records were collected, based on the participants’ age, sex, weight, and daily dietary intake. Of these, 22 samples were missing weight data, resulting in a final valid sample size of 55,678.

### 2.6. Estimation of Dietary Exposure to SCCPs and MCCPs from Chicken Eggs

Based on our previous research methods [34,35], individual exposure levels to CPs from chicken eggs were estimated by multiplying the concentration of CPs in the eggs by individual daily egg consumption, and then correcting for body weight. The Monte Carlo simulation method was used to fit the individual weight and egg consumption data for each of the 55,678 subjects, generating distribution data. The Monte Carlo simulation was run for 10,000 iterations, with one fitting, and the chronic exposure was calculated based on the mean and 95th percentile. The daily exposures to SCCPs and MCCPs were calculated separately, as described in the following formula:

$${Exp}_{i}\;=\;\sum_{i=1}^{n}\frac{F\times Ci}{BWi}$$

where *Exp_i_* represents the dietary exposure to CPs of individual *i* from chicken eggs and their products (μg/kg bw/day); C*_i_* is the consumption of individual *i* from chicken eggs and their products (g/day); F is the mean concentration of CPs in chicken egg (μg/kg); BW*_i_* is the body weight of individual *i* (kg).

### 2.7. Risk Assessment of SCCPs and MCCPs

Due to the lack of toxicological data on CPs and their potential carcinogenicity, it is not possible to derive a health-based guidance value. Instead, the margin of exposure (MOE) approach is used to assess the health risk of CP exposure in humans. In this study, the benchmark dose lower confidence limit at 10% (BMDL_10_) of 2.3 mg/kg bw/day for SCCPs was selected, as it corresponds to an increased incidence of nephritis in male rats. Additionally, the BMDL_10_ of 36 mg/kg bw/day for MCCPs was chosen, which corresponds to an increase in relative kidney weight in both male and female rats [36]. MOE is defined as the ratio of BMDL_10_ to daily dietary exposure. A ratio > 1000 suggests that health risks do not need to be prioritized [36].

### 2.8. Statistical Analysis

Microsoft EXCEL was used to collect consumption data and CP detection data. The concentrations of SCCPs and MCCPs are presented as mean ± standard deviation. Since the concentration of CPs in chicken eggs does not follow a normal distribution, non-parametric tests, including the Kruskal–Wallis test, were used to analyze significant differences in CP levels across different regions. R language (Version 4.0.3) was employed to analyze significant differences, with a significance level set at 0.05 (two-tailed). The average exposure of the population was simulated using @RISK software (Version 8.8.1), and the results are presented as mean and 95% confidence interval (95%CI).

## 3. Results

### 3.1. Occurrence of SCCPs and MCCPs in Chicken Egg Samples

The concentrations of SCCPs and MCCPs are reported by wet weight (ww) in this study. Table 1 shows the detection rates, geometric means, and ranges of ΣSCCPs and ΣMCCPs in chicken egg samples collected from five regions. The detection rate of CPs in all samples of the five regions was 100%. The average concentrations of SCCPs and MCCPs in all samples were 28.4 ng/g ww and 176.5 ng/g ww, respectively, with ranges of 4.7–82.1 ng/g ww for SCCPs and 60.6–410.7 ng/g ww for MCCPs. When comparing samples from different regions, it was found that the level of SCCPs in the Tianjin samples was significantly lower than that in other regions (*p* < 0.001). The average concentration of MCCPs in the Hebei samples was significantly higher than in other regions (*p* < 0.001). This study also compared the SCCPs and MCCPs pollution levels between the southern regions, represented by Jiangxi province, and the northern regions, represented by the other four provinces. The results showed that the average SCCPs concentration (43.2 ± 19.3 ng/g ww) in the southern region was significantly higher than in the northern region (24.7 ± 13.2 ng/g ww, *p* = 0.001). Regarding MCCPs, there was no significant difference between the southern region (148.3 ± 54.1 ng/g ww) and the northern region (183.6 ± 88.4 ng/g ww, *p* = 0.313).

### 3.2. Congener Group Profile of SCCPs and MCCPs in Chicken Egg Samples

**Figure 2:**
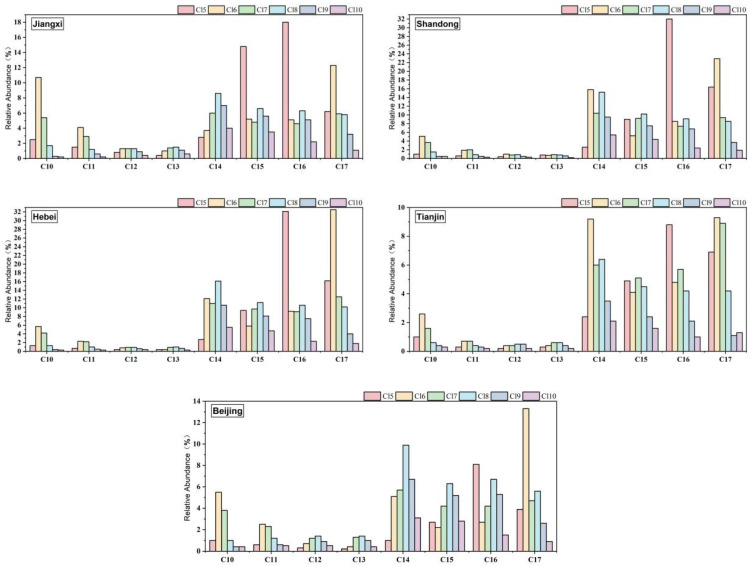
Concentrations of SCCP and MCCP congener groups (ng/g ww) in chicken eggs.

A significant Spearman’s correlation was found between the SCCPs and MCCPs (r = 0.767, *p* = 0.02) in eggs collected from Tianjin. However, no significant correlation was observed in samples from other regions. When analyzing the correlation between egg SCCPs and MCCPs in northern China, a statistically significant correlation was found (r = 0.327, *p* = 0.03), which was likely mainly driven by the data from Tianjin. Further details are provided in Table S3. In this study, 24 SCCP and 24 MCCP congener groups were analyzed in fresh chicken eggs, and the concentrations of each group are shown in Figure 2. For SCCPs, the distribution of species was similar across chicken eggs from different regions, with C_10–11_ Cl _6–7_ being the predominant carbon homolog. In terms of carbon chain length, C_10_-CP had the highest content, accounting for 48%, 46%, 47%, 46%, and 41% of SCCP concentration in chicken egg samples from Jiangxi, Shandong, Hebei, Tianjin, and Beijing, respectively, followed by C_11_-CP, which accounted for 20–26%. Regarding chlorine substitutions, Cl_6_-CP and Cl_7_-CP contributed the most to SCCPs, with contributions ranging from 30 to 39% and from 23 to 30%, respectively. For MCCPs, their contribution to the total CP concentration was much higher than that of SCCPs, accounting for 62–96% of the total CPs. Unlike SCCPs, the distribution characteristics of similar species in MCCPs showed regional variations. In the egg samples from Jiangxi, Shandong, Hebei, Tianjin, and Beijing, a similar abundance of carbon homologs was observed, with the average contributions of C_14_-CP, C_15_-CP, C_16_-CP, and C_17_-CP to the total MCCP concentration being 25%, 21%, 27%, and 27%, respectively. The distribution of chlorine congener groups varied slightly across regions: the contribution rates of Cl_5_-CP, Cl_6_-CP, and Cl_8_-CP were 28%, 18% and 18%, respectively. Cl_5–6_-CP was the dominant chlorine congener group in eggs from Hebei and Shandong, accounting for 47% and 48%, respectively. The low chlorine congener group contributed the most in the samples from Tianjin, with Cl_5–7_-CP accounting for 69% of the MCCP levels. In contrast to other regions, the egg samples from Beijing were dominated by high chlorine homologs, with the contribution rates of Cl_8_-CP, Cl_6_-CP, and Cl_9_-CP being 25%, 20%, and 17%, respectively. Detailed concentration data are provided in Table S4.

### 3.3. Estimates of Daily Intakes of SCCPS and MCCPs in Chicken Eggs

In this study, a total of 55,678 individuals were divided into eight subgroups based on age and sex (3–6, 7–12, 13–17 male, 13–17 female, 18–59 male, 18–59 female, ≥60 male, and ≥60 female), and the daily consumption for each subgroup was calculated separately. We also set up a “consumer-only” group, where every member of the group consumed eggs every day. The average consumption of chicken eggs in the entire population was 30.3 g/d, with the maximum consumption reaching 433.3 g/d (more details can be found in Table S5). Based on individual consumption data and body weight data, we estimated the average levels of exposure to SCCPs and MCCPs in different subgroups. As shown in Table 2, the estimated average levels of SCCP and MCCP exposure from chicken eggs in the Chinese population were 18.3 (95%CI: 0.9) ng/kg bw/d and 118.3 (95%CI: 17.9) ng/kg bw/d, respectively. The exposure levels for high consumption individuals were 53.8 ng/kg bw/d and 339.1 ng/kg bw/d, respectively. The mean exposure levels of SCCPs and MCCPs for each sex–age group ranged from 12.9 (95%CI:0.3) to 56.2 (95%CI: 3.6) ng/kg bw/d and 79.9 (95%CI: 1.6) to 350.9 (95%CI: 16.8) ng/kg bw/d, respectively. Among the sex–age groups, the highest average exposure to SCCPs and MCCPs occurred in the 3–6-year-old group, followed by the 7–12-year-old group, with the lowest exposure found in the ≥60 male group. In the consumer-only group, the average exposure levels of SCCPs and MCCPs were 27.8 (95%CI: 2.4) ng/kg bw/d and 174.1 (95%CI: 24.1) ng/kg bw/d, respectively.

CP pollution levels in different regions will have an important impact on the exposure level of the population. Therefore, we further analyzed the exposure level of the population in different regions, as shown in Table 3. For SCCPs, the average exposure level was highest in Jiangxi and lowest in Tianjin. Regarding MCCPs, Hebei had the highest average exposure, followed by Shandong, with the lowest exposure observed in Tianjin.

### 3.4. Health Risk Assessment of Dietary Intake of SCCPs and MCCPs

We used two MOEs to assess health risks. The MOE calculated based on the average exposure level was labeled as MOE1, while the MOE calculated based on the 95th percentile exposure level was labeled as MOE2. For SCCPs, the MOE1 in different subgroups ranged from 40,925 to 178,295, and the MOE2 ranged from 12,714 to 59,278. The MOE1 for the entire population and the consumer only group were 125,683 and 82,734, respectively, while the MOE2 for these two groups were 35,222 and 42,751, respectively. For MCCPs, the MOE1 in different subgroups ranged from 102,593 to 450,563, and the MOE2 ranged from 31,701 to 145,867. The MOE1 for the entire population and the consumer only group were 304,311 and 206,778, respectively, while the MOE2 for these two groups were 90,794 and 106,163, respectively (Table S6). Health risk attributed to CPs in the entire population from different regions was also calculated (Table S7). For SCCPs, the MOE1 in different regions ranged from 82,734 to 258,427, and the MOE2 ranged from 27,414 to 83,942. For MCCPs, the MOE1 in different regions ranged from 230,474 to 533,333, and the MOE2 ranged from 73,922 to 173,494. The MOE1 and MOE2 for SCCPs were 147,436 and 47,817, and for MCCPs, they were 271,493 and 101,038, respectively, in the northern regions.

## 4. Discussion

With the widespread use of CPs in industry, the resulting environmental pollution has become increasingly severe [1]. The frequency of detecting CPs in the human body is rising, and the associated health impacts deserve serious attention [11,12]. Diet is the primary route of exposure, particularly through fat-rich foods [26]. Chicken eggs are one of the most common food items in the Chinese diet. Although previous studies have detected CPs even in eggs from the Tibetan Plateau, the contamination levels in eggs sold in inland markets remain unclear [27].

The average SCCPs concentration in the study was lower than that in egg mixed samples reported in the sixth national total diet survey in China (64 ng/g ww) [31] and in a study by Dong et al. (46 ng/g ww, duck eggs) [37], which was similar to the study by Ding et al. (24.8 ng/g ww) [25]. It was lower than the average concentrations of SCCPs in chicken and duck egg mixed samples in Korean food surveys (286 ng/g ww) [28] and higher than that in basket egg mixed samples from southern Germany [38] (10.0 ng/g ww) and Belgium (<0.5 ng/g ww) [39]. The average concentration of MCCPs in this study was higher than that reported in egg mixed samples from the sixth national total dietary survey in China (46 ng/g ww) [31], the study of Dong et al. (18 ng/g ww, duck eggs) [37], Ding et al. (17 ng/g ww) [25], southern Germany (4.7 ng/g ww) [38], and Belgium (9 ng/g ww) [39]. Zhou et al. detected SCCPs (428 ng/g ww) and MCCPs (3630 ng/g ww) in farmed chicken eggs from Jiangxi, which were higher than those in our study [27]. Zeng et al. reported levels of SCCPs and MCCPs in free-range eggs collected from an e-waste disposal site in south China, with median levels of SCCPs and MCCPs of 1490 ng/g lipid-weight (lw) and 999 ng/g lw, respectively [40]. The average concentration of SCCPs in this study was also much lower than that in another study with free-range chicken eggs (3400 ng/g lw) in an industrial region in southern China [41]. In addition, the results of this study were higher than those of free-range eggs collected at various sites around waste disposal sites in Tanzania, where median concentrations of SCCPs and MCCPs ranged from ND-370 ng/g lw and 500–900 ng/g lw [42]. The SCCPs/MCCPs ratio ranged from 0.05 to 0.62, indicating higher pollution levels of MCCPs compared to SCCPs. This finding contradicts previous studies and suggests that MCCPs have, to some extent, become industrial substitutes for SCCPs due to regulatory controls on the latter [31,40]. The average concentrations of SCCPs and MCCPs in eggs from Tianjin were relatively low, while the highest average concentrations were detected in eggs from Hebei. This regional disparity may be linked to local industrial activities, as Tianjin has significantly fewer industrial enterprises compared to Hebei and Shandong [43].

Ding et al. reported a significant positive correlation between SCCPs and MCCPs in chicken egg samples from Shandong, which was inconsistent with our findings [25]. The positive correlation between MCCPs and SCCPs may be explained by their similar exposure and/or accumulation behavior [25,29,44]. The analysis of the congener group of SCCPs and MCCPs can provide more information. For SCCPs, C_10–11_ Cl_6–7_ were the main carbon homologs. This finding was consistent with studies by Ding et al. [25], Cui et al. [31], Dong et al. [37], and Haarr et al. [42], but contrasted with results reported by Zhou et al. (C_13_-CP) [27] and Lee et al. (C_12_Cl_8_) [28]. For MCCPs, the balanced distribution of C_14–17_ carbon homologs is not commonly found in food [25]. A similar distribution pattern was observed in a study of black-tailed gull eggs, where the relative abundance of MCCP homologs with longer carbon chains increased from 2012 to 2018, and the major homologs changed from C_15_-CP to C_17_-CP [45]. An increasing trend in the relative abundance of long carbon chain MCCP homologs was also observed in urban and rural Chinese breast milk samples between 2007 and 2017 [46]. Different from the study, C_14_-CP made the most contribution to MCCPs in the studies by Zhou et al. [27], Dong et al. [37], Zeng et al. [40], and Haarr et al. [42]. Compared to other foods, the contamination of chicken eggs with CPs originates from laying hens. Laying hens can be exposed to CPs by consuming contaminated feed or ingesting soil that contains CPs [47]. Dong et al. found that SCCPs (640 ng/g) and MCCPs (78 ng/g) in animal feed materials were higher than in meat and its products. In addition, the congener group profiles of SCCPs and MCCPs were similar to those found in this study [48]. Aamir et al. investigated SCCPs in the topsoil and deep soil layers of agricultural land in China in 2016 and found that the concentration of SCCPs in the topsoil ranged from 39 to 1609 ng/g (dry weight) [49]. Homologs with low chlorination (Cl_5–7_) and short chain lengths (C_10–13_) dominated, which is similar to our study. Due to the presence of a certain amount of fat in chicken eggs, these pollutants are more likely to accumulate, ultimately resulting in the transfer of CPs from feed/soil to laying hens and subsequently to eggs.

The estimated daily intake (EDI) of SCCPs and MCCPs from chicken eggs in the entire population was 18.3 (95%CI: 0.9) ng/kg bw/d and 118.3 (95%CI: 17.9) ng/kg bw/d. Compared with other studies, the EDI of SCCPs in this study was similar to that of Cui et al. (16 ng/kg bw/d) [31], and lower than the median value reported for free-range eggs in adults and children from rural areas of Tibetan plateau (81.6 ng/kg bw/d and 220.2 ng/kg bw/d) [27], and higher than the levels reported by the study by Dong et al. (9.5 ng/kg bw/d) [37]. The EDI of MCCPs in this study was lower than reported for adults and children by Zhou et al. (483.4 ng/kg bw/d and 2911 ng/kg bw/d) [27]. However, it was higher than the MCCP intake levels reported by Cui et al. (8.9 ng/kg bw/d) [31] and Dong et al. (4.2 ng/kg bw/d) [37]. Notably, although the dietary exposure levels of SCCPs and MCCPs vary across different countries and regions, the corresponding MOEs consistently exceed 1000, indicating a relatively low potential health risk under current exposure scenarios.

Our study showed that chicken egg consumption does not pose a health risk associated with CP exposure, but there are some uncertainties and limitations: 1. Although this study covered major provinces in inland regions, the sample representation may be limited. 2. We focused solely on chicken eggs without analyzing the impact of cooking methods. Studies have shown that cooking oil is easily contaminated by CPs, which may lead to an underestimation of exposure risks [50]. 3. The dietary intake risk of the Chinese population is not sufficient from eggs alone. The source of CPs in eggs should be further studied, particularly the pollution in broilers.

## 5. Conclusions

This study investigated the contamination of SCCPs and MCCPs in chicken egg samples from different regions of China. We analyzed the contamination patterns and the composition of related homologs in these samples. Additionally, we assessed dietary exposure to SCCPs and MCCPs through chicken egg consumption among Chinese residents and evaluated the associated health risks. The results indicated that consumption of chicken eggs by Chinese residents does not pose significant health risks related to CPs.

## Figures and Tables

**Table 1:** SCCPs and MCCPs concentrations (ng/g ww) in chicken egg samples.

Sample	Regions	Concentrations of SCCPs			Concentrations of MCCPs			*p*-Value	
		Samples Detected	Mean	Range	Samples Detected	Mean	Range	SCCPs	MCCPs
Chicken egg	Jiangxi	11 (100%)	43.2 ± 19.3	18.6–82.1	11 (100%)	148.3 ± 54.1	77.1–254.3	<0.001 ^a^	<0.001 ^b^
	Shandong	10 (100%)	26.2 ± 9.4	13.2–41.5	10 (100%)	230.1 ± 81.1	80.6–352.6	0.001 ^c^	0.313 ^d^
	Hebei	15 (100%)	27.2 ± 16.7	11.3–80.7	15 (100%)	246.9 ± 69.0	140.3–410.7		
	Tianjin	9 (100%)	13.8 ± 8.0	4.7–32.5	9 (100%)	105.9 ± 26.2	72.2–153.7		
	Beijing	10 (100%)	29.2 ± 9.7	13.2–42.8	10 (100%)	112.0 ± 44.3	60.6–187.9		
	North ^e^	44 (100%)	24.7 ± 13.2	4.7–80.7	44 (100%)	183.6 ± 88.4	60.6–410.7		
Total		55 (100%)	28.4 ± 16.2	4.7–82.1	55 (100%)	176.5 ± 83.5	60.6–410.7		

^a^: Kruskal–Wallis test for SCCP levels among the five provinces. ^b^: Kruskal–Wallis test for MCCP levels among the five provinces. ^c^: Kruskal–Wallis test for SCCP levels between the southern and northern regions. ^d^: Kruskal–Wallis test for MCCP levels between the southern and northern regions. ^e^: Northern region (Shandong, Hebei, Tianjin, and Beijing).

**Table 2:** Exposure levels of CPs from chicken eggs (ng/kg bw/d) in the entire population and across sex–age groups.

Group	SCCPs		MCCPs	
	Mean (95%CI)	P95	Mean (95%CI)	P95
3–6	56.2 ± 3.6	180.9	350.9 ± 16.8	1135.6
7–12	24.5 ± 0.6	92.4	182.9 ± 3.9	576.7
13–17 male	18.1 ± 0.4	55.1	114.0 ± 4.3	352.5
13–17 female	19.2 ± 0.4	58.8	119.0 ± 2.5	362.8
18–59 male	13.0 ± 0.3	39.4	80.6 ± 1.7	246.8
18–59 female	15.3 ± 0.3	47.3	94.4 ± 2.0	284.6
≥60 male	12.9 ± 0.3	38.8	79.9 ± 1.6	247.2
≥60 female	14.1 ± 0.3	42.3	87.1 ± 1.8	268.1
Consumer-only	27.8 ± 2.4	65.3	174.1 ± 24.1	396.5
All	18.3 ± 0.9	53.8	118.3 ± 17.9	339.1

**Table 3:** Chicken egg-derived CP exposure levels (ng/kg bw/d) for the entire population across different regions.

Regions	SCCPs		MCCPs	
	Mean (95%CI)	P95	Mean (95%CI)	P95
Jiangxi	27.8 ± 1.4	83.9	95.2 ± 5.2	286.9
Shandong	16.7 ± 0.7	50.6	114.7 ± 5.0	449.3
Hebei	17.9 ± 2.0	53.3	156.2 ± 6.0	487.0
Tianjin	8.9 ± 0.6	27.4	67.5 ± 2.7	207.5
Beijing	18.4 ± 0.7	56.8	70.4 ± 2.1	212.6
North	15.6 ± 0.7	48.1	132.6 ± 34.5	356.3

## Data Availability

The original contributions presented in this study are included in the article/Supplementary Material. Further inquiries can be directed to the corresponding authors.

## Supplementary Materials

The following supporting information can be downloaded at: https://www.mdpi.com/article/10.3390/toxics14010060/s1, Figure S1: Chemicals structures of CPs; Figure S2: ECNI-MS spectrum of C_10_Cl_6_ (SCCP); Figure S3: ECNI-MS spectrum of C_15_Cl_6_ (MCCP); Figure S4: Total Ion Chromatogram of SCCPs in the standard solutions, analyzed by Comprehensive Two-Dimensional Gas Chromatography–Mass Spectrometry; Figure S5: Total Ion Chromatogram of MCCPs in the standard solutions, analyzed by Comprehensive Two-Dimensional Gas Chromatography–Mass Spectrometry; Figure S6: Total Ion Chromatogram of SCCPs in real samples, analyzed by Comprehensive Two-Dimensional Gas Chromatography–Mass Spectrometry; Figure S7: Total Ion Chromatogram of MCCPs in real samples, analyzed by Comprehensive Two-Dimensional Gas Chromatography–Mass Spectrometry; Table S1: CP technical product list; Table S2: Quantitation and qualification ions used for analyzing short- and medium-chain chlorinated paraffins by mass spectrometry; Table S3: Spearman’s correlations between paired CP sub-groups in different regions; Table S4: The concentrations of SCCPs and MCCPs congener group (ng/g ww) in chicken eggs; Table S5: Average chicken egg consumption in different age-sex groups; Table S6: Health risk attributed to chlorinated paraffins in the whole population and in different sex-age groups; Table S7: Health risk attributed to chlorinated paraffins in whole population from different regions; Text S1: Extraction conditions; Text S2: Instrumental analysis.
