# Supplementary material for: Chlorinated Paraffins in Chicken Eggs from Five Regions in China and Dietary Exposure Health Risk Assessment
Source: Toxics. 2026 Jan 8;14(1):60. doi: 10.3390/toxics14010060 (PMC12845650; doi:10.3390/toxics14010060)
Supplement: Supplementary file 1 [file toxics-14-00060-s001.zip › toxics-4040325-supplementary.pdf]

Text S1

Extraction conditions: Temperature of 100℃, pressure of 1.03×10^4 kPa, heating time of 5 minutes, static extraction for 10 minutes, flushing volume of 60%, N_2 purging time of 60 seconds, and three cycles.

Text S2

Instrumental analysis.: A non-polar DB-5MS (30 m × 0.25 mm × 0.25 μm; Agilent Technologies) was used as the first-dimensional column, and a mid-polar BPX-50 (1 m × 0.10 mm × 0.10 μm; SGE, Melbourne, Australia) was used as the second-dimensional column. The instrument settings were as follows: the initial oven temperature was set at 100 ℃ and held for 1 min, then increased to 140℃ at a rate of 10 ℃/min, followed by a ramp to 310 ℃ at 1.5 ℃/min, and finally held at 310 ℃ for 5 min. A splitless injection mode was used with an injection volume of 1 μL. The injector temperature was maintained at 280 ℃. The carrier gas was helium (purity: 99.999%) at a constant flow rate of 0.8 mL/min, with a modulation period of 7 s. The ion source and transfer line temperatures were set at 200 ℃ and 280 ℃, respectively.

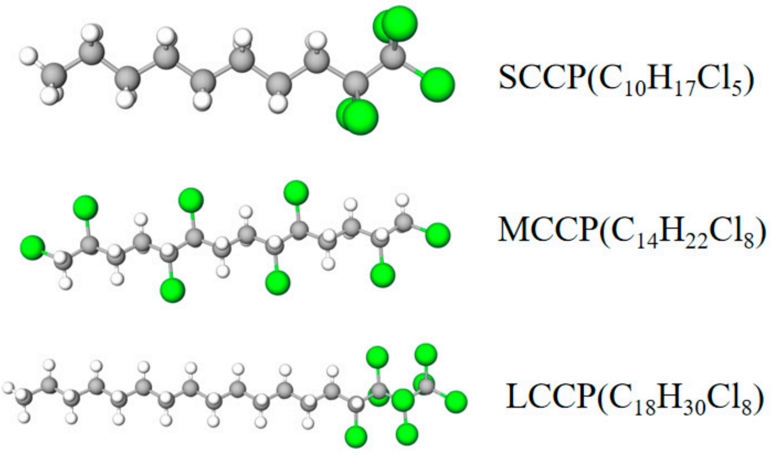

Figure S1 Chemicals structures of CPs.

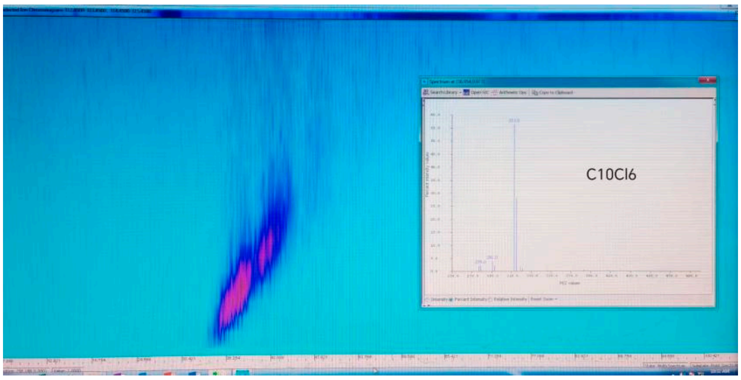

Figure S2 ECNI-MS spectrum of C10Cl6 (SCCP).

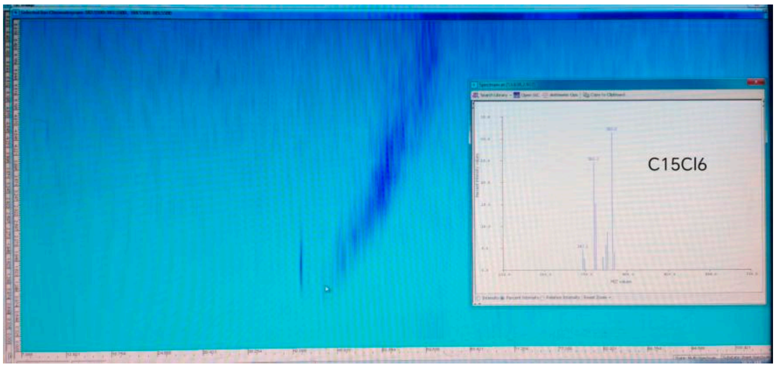

Figure S3 ECNI-MS spectrum of $C_{15}Cl_6$ (MCCP).

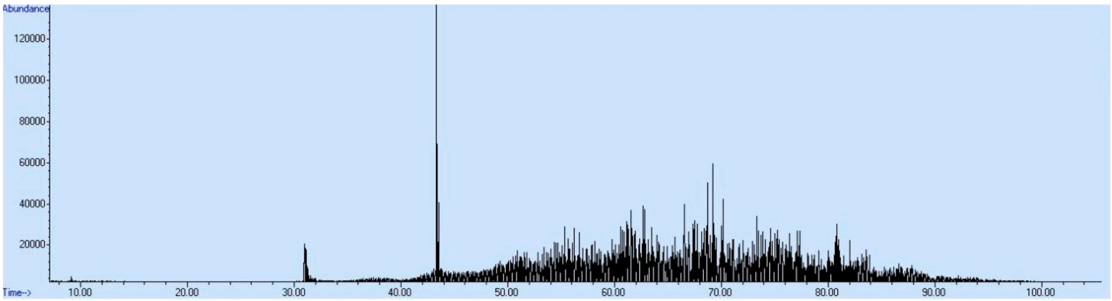

Figure S4 Total Ion Chromatogram of SCCPs in the standard solutions, analyzed by Comprehensive Two-Dimensional Gas Chromatography–Mass Spectrometry.

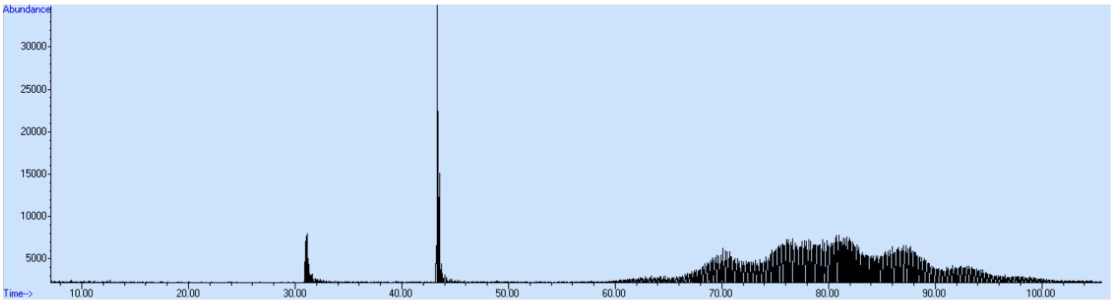

Figure S5 Total Ion Chromatogram of MCCPs in the standard solutions, analyzed by Comprehensive Two-Dimensional Gas Chromatography–Mass Spectrometry.

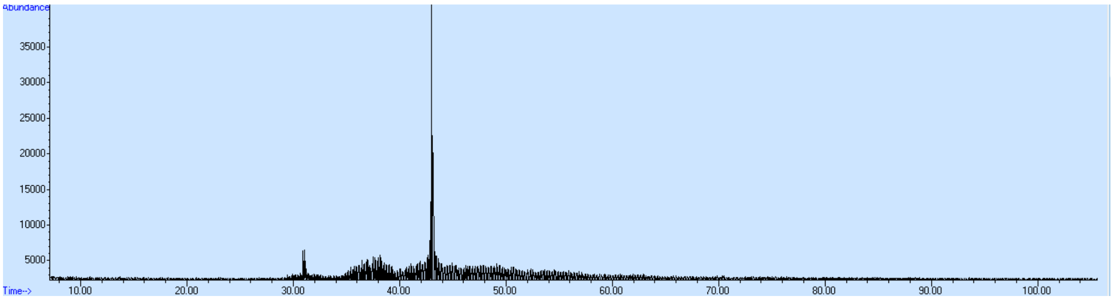

Figure S6 Total Ion Chromatogram of SCCPs in real samples, analyzed by Comprehensive Two-Dimensional Gas Chromatography–Mass Spectrometry.

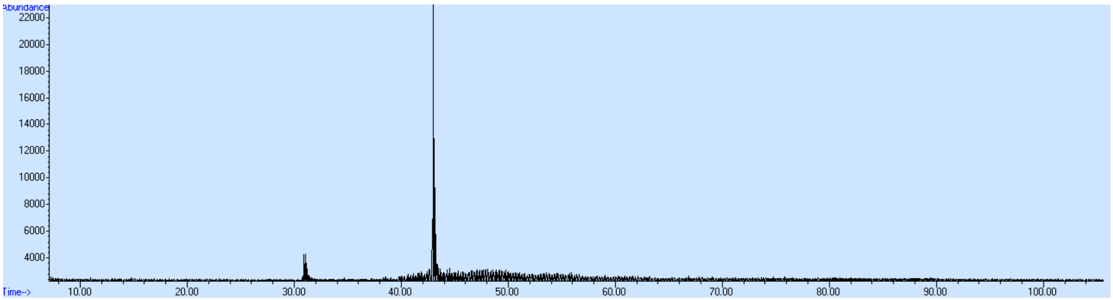

Figure S7 Total Ion Chromatogram of MCCPs in real samples, analyzed by Comprehensive Two-Dimensional Gas Chromatography–Mass Spectrometry.

Table S1 CP technical product list.

**Standards**	**Manufacturer**	**Product Code**	**CAS**
Chloroparaffin C10-C13, 51,5% Cl 100 μg/mL in Cyclohexane	Dr. Ehrenstorfer	DRE-X23105100CY	85535-84-8
Chloroparaffin C10-C13, 55,5% Cl 100 μg/mL in Cyclohexane		DRE-X23105500CY	85535-84-8
Chloroparaffin C10-C13, 63% Cl 100 μg/mL in Cyclohexane		DRE-X23106300CY	85535-84-8
Chloroparaffin C14-C17 42% Cl 100 μg/mL in Cyclohexane		DRE-X23144200CY	85535-85-9
Chloroparaffin C14-C17 52% Cl 100 μg/mL in Cyclohexane		DRE-X23145200CY	85535-85-9
Chloroparaffin C14-C17 57% Cl 100 μg/mL in Cyclohexane		DRE-X23145700CY	85535-85-9
ε-hexachlorocyclohexane (10 ng/μL)		DRE-L14073000CY	58-89-9
**Other Reagents**	**Manufacturer**	**Purity**	**CAS**
n-hexane	JT Baker	≥95%	110-54-3
acetone		≥99.7%	67-64-1

cyclohexane		≥99.5%	110-82-7
dichloromethane		≥99.8%	75-09-2
methanol		≥99.5%	67-56-1
acetone	Sinopharm Chemical Reagent	≥99.5%	67-64-1
cyclohexane		≥99.7%	110-82-7
13C10-trans-chlordane	Cambridge Isotope Laboratories	99%	1262969-05-0

Table S2 Quantitation and qualification ions used for analyzing short- and medium-chain chlorinated paraffins by mass spectrometry.

Isomer formula/(n,z[a])	Quantitation ion	Ion formation	Qualification ion	Ion formation
10,5	279.00	$C_{10}H_{17}{}^{35}Cl_3{}^{37}Cl$	277.00	$C_{10}H_{17}{}^{35}Cl_4$
10,6	312.95	$C_{10}H_{16}{}^{35}Cl_4{}^{37}Cl$	314.95	$C_{10}H_{16}{}^{35}Cl_3{}^{37}Cl_2$
10,7	346.95	$C_{10}H_{15}{}^{35}Cl_5{}^{37}Cl$	348.90	$C_{10}H_{15}{}^{35}Cl_4{}^{37}Cl_2$
10,8	380.90	$C_{10}H_{14}{}^{35}Cl_6{}^{37}Cl$	382.90	$C_{10}H_{14}{}^{35}Cl_5{}^{37}Cl_2$
10,9	416.85	$C_{10}H_{13}{}^{35}Cl_6{}^{37}Cl_2$	414.85	$C_{10}H_{13}{}^{35}Cl_7{}^{37}Cl$
10,10	450.80	$C_{10}H_{12}{}^{35}Cl_7{}^{37}Cl_2$	448.80	$C_{10}H_{12}{}^{35}Cl_8{}^{37}Cl$
11,5	293.00	$C_{11}H_{19}{}^{35}Cl_3{}^{37}Cl$	291.00	$C_{11}H_{19}{}^{35}Cl_4$
11,6	327.00	$C_{11}H_{18}{}^{35}Cl_4{}^{37}Cl$	329.00	$C_{11}H_{18}{}^{35}Cl_3{}^{37}Cl_2$
11,7	360.95	$C_{11}H_{17}{}^{35}Cl_5{}^{37}Cl$	362.95	$C_{11}H_{17}{}^{35}Cl_4{}^{37}Cl_2$
11,8	394.90	$C_{11}H_{16}{}^{35}Cl_6{}^{37}Cl$	396.90	$C_{11}H_{16}{}^{35}Cl_5{}^{37}Cl_2$
11,9	430.85	$C_{11}H_{15}{}^{35}Cl_6{}^{37}Cl_2$	428.85	$C_{11}H_{15}{}^{35}Cl_7{}^{37}Cl$
11,10	464.80	$C_{11}H_{14}{}^{35}Cl_7{}^{37}Cl_2$	462.85	$C_{11}H_{14}{}^{35}Cl_8{}^{37}Cl$
12,5	307.05	$C_{12}H_{21}{}^{35}Cl_3{}^{37}Cl$	305.05	$C_{12}H_{21}{}^{35}Cl_4$
12,6	341.00	$C_{12}H_{20}{}^{35}Cl_4{}^{37}Cl$	343.00	$C_{12}H_{20}{}^{35}Cl_3{}^{37}Cl_2$
12,7	374.95	$C_{12}H_{19}{}^{35}Cl_5{}^{37}Cl$	376.95	$C_{12}H_{19}{}^{35}Cl_4{}^{37}Cl_2$
12,8	408.90	$C_{12}H_{18}{}^{35}Cl_6{}^{37}Cl$	410.90	$C_{12}H_{18}{}^{35}Cl_5{}^{37}Cl_2$
12,9	444.90	$C_{12}H_{17}{}^{35}Cl_6{}^{37}Cl_2$	442.90	$C_{12}H_{17}{}^{35}Cl_7{}^{37}Cl$
12,10	478.85	$C_{12}H_{16}{}^{35}Cl_7{}^{37}Cl_2$	476.85	$C_{12}H_{16}{}^{35}Cl_8{}^{37}Cl$
13,5	321.05	$C_{13}H_{23}{}^{35}Cl_3{}^{37}Cl$	319.05	$C_{13}H_{23}{}^{35}Cl_4$
13,6	355.00	$C_{13}H_{22}{}^{35}Cl_4{}^{37}Cl$	357.00	$C_{13}H_{22}{}^{35}Cl_3{}^{37}Cl_2$
13,7	388.95	$C_{13}H_{21}{}^{35}Cl_5{}^{37}Cl$	390.95	$C_{13}H_{21}{}^{35}Cl_4{}^{37}Cl_2$
13,8	422.95	$C_{13}H_{20}{}^{35}Cl_6{}^{37}Cl$	424.95	$C_{13}H_{20}{}^{35}Cl_5{}^{37}Cl_2$
13,9	458.90	$C_{13}H_{19}{}^{35}Cl_6{}^{37}Cl_2$	456.90	$C_{13}H_{19}{}^{35}Cl_7{}^{37}Cl$
13,10	492.85	$C_{13}H_{18}{}^{35}Cl_7{}^{37}Cl_2$	490.85	$C_{13}H_{18}{}^{35}Cl_8{}^{37}Cl$
14,5	335.05	$C_{14}H_{25}{}^{35}Cl_3{}^{37}Cl$	333.05	$C_{14}H_{25}{}^{35}Cl_4$
14,6	369.05	$C_{14}H_{24}{}^{35}Cl_4{}^{37}Cl$	371.05	$C_{14}H_{24}{}^{35}Cl_3{}^{37}Cl_2$

14,7	403.00	$C_{14}H_{23}{}^{35}Cl_5{}^{37}Cl$	405.00	$C_{14}H_{23}{}^{35}Cl_4{}^{37}Cl_2$
14,8	436.95	$C_{14}H_{22}{}^{35}Cl_6{}^{37}Cl$	438.95	$C_{14}H_{22}{}^{35}Cl_5{}^{37}Cl_2$
14,9	472.90	$C_{14}H_{21}{}^{35}Cl_6{}^{37}Cl_2$	470.90	$C_{14}H_{21}{}^{35}Cl_7{}^{37}Cl$
14,10	506.85	$C_{14}H_{20}{}^{35}Cl_7{}^{37}Cl_2$	504.85	$C_{14}H_{20}{}^{35}Cl_8{}^{37}Cl$
15,5	349.10	$C_{15}H_{27}{}^{35}Cl_3{}^{37}Cl$	347.10	$C_{15}H_{27}{}^{35}Cl_4$
15,6	383.05	$C_{15}H_{26}{}^{35}Cl_4{}^{37}Cl$	385.05	$C_{15}H_{26}{}^{35}Cl_3{}^{37}Cl_2$
15,7	417.00	$C_{15}H_{25}{}^{35}Cl_5{}^{37}Cl$	419.00	$C_{15}H_{25}{}^{35}Cl_4{}^{37}Cl_2$
15,8	450.95	$C_{15}H_{24}{}^{35}Cl_6{}^{37}Cl$	452.95	$C_{15}H_{24}{}^{35}Cl_5{}^{37}Cl_2$
15,9	486.90	$C_{15}H_{23}{}^{35}Cl_6{}^{37}Cl_2$	484.95	$C_{15}H_{23}{}^{35}Cl_7{}^{37}Cl$
15,10	520.90	$C_{15}H_{22}{}^{35}Cl_7{}^{37}Cl_2$	518.90	$C_{15}H_{22}{}^{35}Cl_8{}^{37}Cl$
16,5	363.10	$C_{16}H_{29}{}^{35}Cl_3{}^{37}Cl$	361.10	$C_{16}H_{29}{}^{35}Cl_4$
16,6	397.05	$C_{16}H_{28}{}^{35}Cl_4{}^{37}Cl$	399.05	$C_{16}H_{28}{}^{35}Cl_3{}^{37}Cl_2$
16,7	431.00	$C_{16}H_{27}{}^{35}Cl_5{}^{37}Cl$	433.00	$C_{16}H_{27}{}^{35}Cl_4{}^{37}Cl_2$
16,8	465.00	$C_{16}H_{26}{}^{35}Cl_6{}^{37}Cl$	467.00	$C_{16}H_{26}{}^{35}Cl_5{}^{37}Cl_2$
16,9	500.95	$C_{16}H_{25}{}^{35}Cl_6{}^{37}Cl_2$	498.95	$C_{16}H_{25}{}^{35}Cl_7{}^{37}Cl$
16,10	534.90	$C_{16}H_{24}{}^{35}Cl_7{}^{37}Cl_2$	532.90	$C_{16}H_{24}{}^{35}Cl_8{}^{37}Cl$
17,5	377.10	$C_{17}H_{31}{}^{35}Cl_3{}^{37}Cl$	375.10	$C_{17}H_{31}{}^{35}Cl_4$
17,6	411.10	$C_{17}H_{30}{}^{35}Cl_4{}^{37}Cl$	413.05	$C_{17}H_{30}{}^{35}Cl_3{}^{37}Cl_2$
17,7	445.05	$C_{17}H_{29}{}^{35}Cl_5{}^{37}Cl$	447.05	$C_{17}H_{29}{}^{35}Cl_4{}^{37}Cl_2$
17,8	479.00	$C_{17}H_{28}{}^{35}Cl_6{}^{37}Cl$	481.00	$C_{17}H_{28}{}^{35}Cl_5{}^{37}Cl_2$
17,9	514.95	$C_{17}H_{27}{}^{35}Cl_6{}^{37}Cl_2$	512.95	$C_{17}H_{27}{}^{35}Cl_7{}^{37}Cl$
17,10	548.90	$C_{17}H_{26}{}^{35}Cl_7{}^{37}Cl_2$	546.90	$C_{17}H_{26}{}^{35}Cl_8{}^{37}Cl$

[a] n is the number of carbon atoms and z is the number of chlorine atoms.

Table S3. Spearman's correlations between paired CP sub-groups in different regions.

Regions	SCCPs-MCCPs	
	r	*P*-value
Jiangxi	0.409	0.21
Shandong	0.176	0.63
Hebei	0.059	0.84
Tianjin	**0.767**	**0.02**
Beijing	0.091	0.81
North	**0.327**	**0.03**
All	0.223	0.10

Correlations with statistical significance ($p < 0.05$) are highlighted in bold.

Table S4. The concentrations of SCCP and MCCP congener groups (ng/g ww) in

chicken eggs.

SCCPs	Jiangxi	Shandong	Hebei	Tianjin	Beijing	MCCPs	Jiangxi	Shandong	Hebei	Tianjin	Beijing
$C_{10}Cl_5$	2.5	1.0	1.3	1.0	1.0	$C_{14}Cl_5$	2.8	2.6	2.7	2.4	1.0
$C_{10}Cl_6$	10.7	5.1	5.7	2.6	5.5	$C_{14}Cl_6$	3.7	15.8	12.1	9.2	5.1
$C_{10}Cl_7$	5.4	3.7	4.2	1.6	3.8	$C_{14}Cl_7$	6.0	10.4	11.0	6.0	5.7
$C_{10}Cl_8$	1.7	1.5	1.3	0.6	1.0	$C_{14}Cl_8$	8.6	15.2	16.1	6.4	9.9
$C_{10}Cl_9$	0.3	0.5	0.4	0.4	0.4	$C_{14}Cl_9$	7.0	9.5	10.6	3.5	6.7
$C_{10}Cl_{10}$	0.2	0.5	0.3	0.3	0.4	$C_{14}Cl_{10}$	4.0	5.4	5.5	2.1	3.1
**$\sum C_{10}$**	**20.8**	**12.3**	**13.2**	**6.5**	**12.1**	**$\sum C_{14}$**	**32.1**	**58.9**	**58.0**	**29.6**	**31.5**
$C_{11}Cl_5$	1.5	0.6	0.7	0.3	0.6	$C_{15}Cl_5$	14.8	9.0	9.4	4.9	2.7
$C_{11}Cl_6$	4.1	1.9	2.3	0.7	2.5	$C_{15}Cl_6$	5.2	5.2	5.8	4.1	2.2
$C_{11}Cl_7$	2.9	2.0	2.2	0.7	2.3	$C_{15}Cl_7$	4.8	9.2	9.7	5.1	4.2
$C_{11}Cl_8$	1.2	0.9	1.0	0.4	1.2	$C_{15}Cl_8$	6.6	10.2	11.2	4.5	6.3
$C_{11}Cl_9$	0.6	0.5	0.5	0.3	0.6	$C_{15}Cl_9$	5.6	7.5	8.1	2.4	5.2
$C_{11}Cl_{10}$	0.2	0.3	0.3	0.2	0.5	$C_{15}Cl_{10}$	3.5	4.4	4.7	1.6	2.8
**$\sum C_{11}$**	**10.5**	**6.2**	**7.0**	**2.6**	**7.7**	**$\sum C_{15}$**	**40.5**	**45.5**	**48.9**	**22.6**	**23.4**
$C_{12}Cl_5$	0.8	0.4	0.4	0.2	0.3	$C_{16}Cl_5$	18.0	32.0	32.1	8.8	8.1
$C_{12}Cl_6$	1.3	1.0	0.8	0.4	0.7	$C_{16}Cl_6$	5.1	8.5	9.2	4.8	2.7
$C_{12}Cl_7$	1.3	0.8	0.9	0.4	1.2	$C_{16}Cl_7$	4.6	7.4	9.1	5.7	4.2
$C_{12}Cl_8$	1.3	0.9	0.9	0.5	1.4	$C_{16}Cl_8$	6.3	9.1	10.6	4.2	6.7
$C_{12}Cl_9$	0.9	0.5	0.6	0.5	0.9	$C_{16}Cl_9$	5.1	6.8	7.5	2.1	5.3
$C_{12}Cl_{10}$	0.4	0.3	0.4	0.2	0.5	$C_{16}Cl_{10}$	2.2	2.4	2.3	1.0	1.5
**$\sum C_{12}$**	**6.0**	**3.9**	**4.0**	**2.2**	**5.0**	**$\sum C_{16}$**	**41.3**	**66.2**	**70.8**	**26.6**	**28.5**
$C_{13}Cl_5$	0.4	0.8	0.4	0.3	0.2	$C_{17}Cl_5$	6.2	16.4	16.2	6.9	3.9
$C_{13}Cl_6$	1.0	0.7	0.4	0.4	0.4	$C_{17}Cl_6$	12.3	22.9	32.5	9.3	13.3
$C_{13}Cl_7$	1.4	0.9	0.9	0.6	1.3	$C_{17}Cl_7$	5.9	9.4	12.5	8.9	4.7
$C_{13}Cl_8$	1.5	0.8	1.0	0.6	1.4	$C_{17}Cl_8$	5.8	8.5	10.2	4.2	5.6
$C_{13}Cl_9$	1.1	0.6	0.7	0.4	1.0	$C_{17}Cl_9$	3.2	3.7	4.0	1.1	2.6
$C_{13}Cl_{10}$	0.6	0.2	0.3	0.2	0.4	$C_{17}Cl_{10}$	1.1	1.9	1.8	1.3	0.9
**$\sum C_{13}$**	**6.0**	**4.0**	**3.7**	**2.5**	**4.7**	**$\sum C_{17}$**	**34.5**	**62.8**	**77.2**	**31.7**	**31.0**

Table S5. Average chicken egg consumption in different age–sex groups.

Group	N	Daily consumption (g/d)					
		Mean	P50	P90	P95	P97.5	Max
3-6	3359	33.0	26.7	56.7	87.3	101.4	240.7
7-12	4785	32.5	26.7	73.3	88.3	90.0	240.0
13-17 male	1156	33.8	28.7	75.7	93.3	110.0	238.3
13-17 female	1101	32.3	26.7	65.2	90.0	110.0	183.3
18-59 male	17211	30.5	21.7	81.4	90.0	106.7	433.3
18-59 female	19227	29.9	21.0	70.0	86.7	103.3	343.3
≥60 male	4342	28.4	20.0	77.0	88.3	106.5	243.3
≥60 female	4497	26.9	20.0	69.8	83.3	97.3	180.0
Consumers	39602	42.5	35.3	80.0	98.3	113.3	433.3

All	55678	30.3	21.7	75.2	88.3	104.3	433.3

Table S6. Health risk attributed to chlorinated paraffins in the whole population and in different sex–age groups.

Group	SCCPs		MCCPs	
	MOE1	MOE2	MOE1	MOE2
3-6	40925	12714	102593	31701
7-12	93878	24892	196829	62424
13-17 male	127072	41742	315789	102128
13-17 female	119792	39116	302521	99228
18-59 male	176923	58376	446650	145867
18-59 female	150327	48626	381356	126493
≥60 male	178295	59278	450563	145631
≥60 female	163121	54374	413318	134278
Consumers	82734	35222	206778	90794
All	125683	42751	304311	106163

MOE1 for average daily intake.
MOE2 for P95 daily intake.

Table S7. Health risk attributed to chlorinated paraffins in whole population from different regions.

Regions	SCCPs		MCCPs	
	MOE1	MOE2	MOE1	MOE2
Jiangxi	82734	27414	378151	125479
Shandong	137725	45455	313862	80125
Hebei	128492	43152	230474	73922
Tianjin	258427	83942	533333	173494
Beijing	125000	40493	511364	169332
North	147436	47817	271493	101038

MOE1 for average daily intake.
MOE2 for P95 daily intake.
